# Automatic classification of protein structures using low-dimensional structure space mappings

**DOI:** 10.1186/1471-2105-15-S2-S1

**Published:** 2014-01-24

**Authors:** Daniel Asarnow, Rahul Singh

**Affiliations:** 1 Department of Computer Science, San Francisco State University, 1600 Holloway Ave, San Francisco, CA, 94132, USA; 2 Center for Discovery and Innovation in Parasitic Diseases, University of California, San Francisco, San Francisco, CA, USA

## Abstract

**Background:**

Protein function is closely intertwined with protein structure. Discovery of meaningful structure-function relationships is of utmost importance in protein biochemistry and has led to creation of high-quality, manually curated classification databases, such as the gold-standard SCOP (Structural Classification of Proteins) database. The SCOP database and its counterparts such as CATH provide a detailed and comprehensive description of the structural and evolutionary relationships of the proteins of known structure and are widely employed in structural and computational biology. Since manual classification is both subjective and highly laborious, automated classification of novel structures is increasingly an active area of research. The design of methods for automated structure classification has been rendered even more important since the recent past, due to the explosion in number of solved structures arising out of various structural biology initiatives.

In this paper we propose an approach to the problem of structure classification based on creating and tessellating low dimensional maps of the protein structure space (MPSS). Given a set of protein structures, an MPSS is a low dimensional embedding of structural similarity-based distances between the molecules. In an MPSS, a group of proteins (such as all the proteins in the PDB or sub-samplings thereof) under consideration are represented as point clouds and structural relatedness maps to spatial adjacency of the points. In this paper we present methods and results that show that MPSS can be used to create tessellations of the protein space comparable to the clade systems within SCOP. Though we have used SCOP as the gold standard, the proposed approach is equally applicable for other structural classifications.

**Methods:**

In the proposed approach, we first construct MPSS using pairwise alignment distances obtained from four established structure alignment algorithms (CE, Dali, FATCAT and MATT). The low dimensional embeddings are next computed using an embedding technique called multidimensional scaling (MDS). Next, by using the remotely homologous Superfamily and Fold levels of the hierarchical SCOP database, a distance threshold is determined to relate adjacency in the low dimensional map to functional relationships. In our approach, the optimal threshold is determined as the value that maximizes the total true classification rate vis-a-vis the SCOP classification. We also show that determining such a threshold is often straightforward, once the structural relationships are represented using MPSS.

**Results and conclusion:**

We demonstrate that MPSS constitute highly accurate representations of protein fold space and enable automatic classification of SCOP Superfamily and Fold-level relationships. The results from our automatic classification approach are remarkably similar to those found in the distantly homologous Superfamily level and the quite remotely homologous Fold levels of SCOP. The significance of our results are underlined by the fact that most automated methods developed thus far have only managed to match the closest-homology Family level of the SCOP hierarchy and tend to differ considerably at the Superfamily and Fold levels. Furthermore, our research demonstrates that projection into a low-dimensional space using MDS constitutes a superior noise-reducing transformation of pairwise distances than do the variety of probability- and alignment-length-based transformations currently used by structure alignment algorithms.

## Background

The discovery of functionally meaningful, structural relationships between proteins is of utmost importance in protein biochemistry. Such relationships constitute the basis for organizational efforts in structural bioinformatics such as the CATH and SCOP databases [1,2]. CATH and SCOP both classify protein structures as members of specific clades defined by a hierarchy of specific biological relationships, but differ significantly in methodology. While both databases use a combination of automatic and manual analysis, CATH relies much more on automatic steps while SCOP is primarily based on expert knowledge. Further differences are apparent in the definition of the hierarchies. Simplistically, CATH is more "structural" while SCOP places more emphasis on evolutionary or functional relationships [3].

The "Superfamily" level of SCOP is especially interesting, because it represents remote homologies implying shared function or evolutionary origin but which are difficult or impossible to detect using sequence information alone. Attempts to automate the functional and evolutionary classification of protein structures have thus developed a natural focus on measures of structural similarity determined by geometric alignment of pairs of protein structures. As a corollary, manually curated databases such as SCOP have frequently been used to provide a ground truth or gold standard for automated identification of structurally and functionally related sets of structures. More recently, the explosion of known, but un-annotated, structures which has followed the advent of structural genomics has underlined both the difficulty and the importance of constructing a holistic description of protein architecture capable of reliably recognizing even very remote types of structural homology.

A highly correlated research thrust begun in the early days of structural biology, when only a few thousand structures had been solved [4,5], focuses on the development of grand views of the "universe" of known protein structures. In such a formulation, every protein structure is envisioned as a point in an abstract, high dimensional fold space or protein structure space (PSS). A number of different representations of PSS have been put forward. These have included representations based on graphical models [6] as well as approximate vector representations of individual structures (similar to structure keys for small molecules) [7,8] but have more commonly been based on pairwise (dis)similarities between proteins, as measured using algorithms for pairwise structure alignment [9-11]. A related approach has been the use of pairwise distances to compute explicit spatial representations of the PSS in terms of coordinates in a reduced Euclidean space [5,12-14]. In these works explicit representations of PSS are constructed by embedding intermolecular distances in a low dimensional space in a manner that is injective and minimally distorts inter-structure dissimilarity information. Such embeddings, referred to as maps of protein structure space (MPSS), may carry a number of advantages over PSS representations based solely on pairwise distances. For example, because MPSS are constructed so as to maximize the mutual consistency of relationships between all pairs of proteins simultaneously, they exhibit greater sensitivity to remote homology than can be obtained using pairwise distance distributions alone. Embedding methods that have been used for MPSS include correspondence analysis using reciprocal averaging [5] as well as principle component analysis [8], but multidimensional scaling (MDS) has been the most widely used approach and is most conducive to the notion of MPSS as described above, because MDS attempts to directly approximate pairwise distances within an explicit coordinate configuration.

### Problem formulation

We seek to investigate the expressive capabilities of MPSS by clustering inter-molecular similarity information presented through such representations and comparing the results with known classification schemes. The observation that MDS may confer an advantage over pairwise alignment distances for annotation inference was made previously in the context of Gene Ontology [12] and was recently corroborated by us in experiments in automatic classification of remotely homologous relationships from CATH and SCOP [13]. In that work we examined the influence of certain critical meta-parameters on construction of MPSS using MDS, including choice of the underlying protein alignment method and particular implementation of MDS. We also demonstrated that one of the major advantages of MPSS lies in representing groups of related proteins which are highly structurally diverse. However, we did not investigate the effects of probability- or length-adjustment of alignment scores, something which is done by different alignment algorithms in a unique manner. The behavior of MPSS with respect to the dimensionality of the representation was also not explored, as the MPSS were restricted to three dimensions to permit visual interpretation. The explicit coordinate representation embodied by MPSS lends itself to addressing fundamental questions relating to the dominant folding pathways, evolutionary processes, and physical constraints that define the structure-function relationship. We can investigate if functional characteristics map to specific regions of the PSS and vice-versa, as well as the distribution of molecules in this space. Such questions are worthy, and MPSS offer a unique approach in the search for their answers, but prior to further work in answering such general questions, it would be prudent to probe the response of MPSS to the parameters named above. Additionally, the usefulness of MPSS for automatic or partially automatic partitioning of PSS, as opposed to recognition of structure-function relationships has not been studied. Both of these tasks are undertaken in this work.

By design, all of the analysis techniques applied below treat distance matrices rather than point configurations. This approach allows both coordinate and distance based PSS representations to be evaluated within the same framework; combining low-dimensional projection with computation of pairwise distances permits MPSS construction to be thought of as a distance transformation method analogous to those used to calculate alignment probability scores. In addition to the CE, Dali and FATCAT algorithms used previously, we also consider both pairwise and MPSS distances derived using MATT, a recent flexible aligner which has been shown to effectively capture remote homologies in SCOP [11]. The parameters of alignment method (4 alternatives), score transformation (2 alternatives) and variation of MDS algorithm (2 alternatives) yield 24 unique PSS representations including 8 sets of pairwise distances as well as 16 sets of MPSS, each of which is computed using eight discrete dimensionalities ranging from 3 to 120. All of these PSS representations are validated against Superfamily and Fold level homologies by using them as predictors of shared annotation for protein pairs, allowing the questions of parameterization and relative classification performance to be answered directly. For a few highly accurate representations, we directly compare the entire SCOP Superfamily and Fold clade systems to the tessellation of the PSS produced by a "SCOP independent" clustering method. Such methods have been defined in the literature as those not using the existing SCOP hierarchy, although they may have a threshold or cluster number parameter which *is *tuned against SCOP [10,11]. We use an agglomerative, hierarchical method first used in conjunction with pairwise MATT probability scores [11], which uses a distance threshold obtained during measurement of classification accuracy to perform a maximum-linkage clustering of a dendrogram constructed by neighbor-joining. The primary contributions of this work include:

• The first analysis known to us of clustering within MPSS. Specifically, we show that clustering of MPSS distances leads to results more similar to existing SCOP clade systems than do pairwise distances, using a parameterized clustering algorithm which tries to account for varying diversity between clades using a hierarchical approach.

• Investigation of parameters critical to the construction of MPSS. Importantly, we show that there is a range of dimensionalities for which MPSS outperform any of the pairwise distances considered here. We also find that alignment score transformations proposed in the literature provide no consistent benefit to performance.

• A systematic approach to the probability and length adjustment of alignment scores. Currently, an *ad hoc *collection of transformations unique to each aligner are used, relying on 1 to 3 free parameters. MPSS represent a new score transformation which 1) outperforms those used by the aligners themselves and 2) has only a single free parameter (dimensionality) which is insensitive enough that a single value can be used effectively for all alignment algorithms.

### Data set

Due both to the large number of known protein structures - more than 93,000 at the time of this writing, as well as the computational expense of pairwise structure alignment, it is not possible to exhaustively explore PSS. Furthermore, some of these structures are highly homologous. As such, it is necessary to subsample the known proteins, to obtain representative data set(s) for which all-pairs alignments may be completed with a reasonable amount of time and resources. The manner of this sampling constitutes an essential parameter when analyzing PSS. Previous large-scale investigations of PSS have employed either sequence clustering of the PDB targeting predetermined levels of sequence identity, ranging from 25% to 80% [12,11,15,10], a combination of sequence- and structure-based clustering designed to result in unique fold structures [9], or the complete set of SCOP domains [8]. The sizes of all these sets range from around 500 to around 30,000. Given that our aim is to globally characterize PSS, with a focus on large structure families, we choose the Nov. 2008 release of PDBSelect25, a list of some 4,000 non-redundant protein structures obtained by clustering the PDB at a 25% identity level [16]. The set contains a sufficient number of sufficiently disparate structures to represent the extents of the protein universe, while remaining small enough that exhaustive pairwise alignment using multiple structure alignment algorithms remains feasible. The low redundancy of the data set is crucial given that remote homologies which are difficult to detect are the subject of such heightened interest. If relatively greater redundancy is included in the data set "easy" homologies are likely to dominate, which may lead to overestimation of classification accuracy in general. In the chosen data set there are 1,180 distinct Superfamilies represented by 3,967 chains, 3,744 of which map to a single SCOP domain. The extent of structure space covered by these structures is comparable to that which was covered by the set of 10,018 domains clustered at 80% sequence identity which was used in [11]. Those domains contained 1,656 Superfamilies, 953 of which also occur in our data.

## Methods

### Protein distances

The distance in structure space between individual pairs of proteins can be determined via pairwise alignment of protein structures. Inasmuch as different algorithms for approximate solution of this NP-Complete problem disagree about the relative similarity of various proteins, they can be expected to lead to divergent representations of PSS. Similarly, agreement between multiple methods would suggest that results are biologically accurate. Of the large number of such algorithms which have been described in the literature, we select four well-known examples: CE [17], Dali [18], FATCAT [19] and MATT [20]. These methods represent radically divergent approaches to the structure alignment problem. Dali is unique in that it searches for similar subsets of protein interatomic distances. CE, FATCAT and MATT align structures directly, producing gapped alignments by optimally chaining aligned fragment pairs (AFP) with dynamic programming, but under different assumptions about protein structure itself. While CE models proteins as rigid bodies, FATCAT and MATT both permit a degree of flexibility within aligned structures. The type of flexibilities modeled by the two algorithms, however, are quite different. FATCAT permits twists between pairs of rigidly aligned fragments, while simultaneously minimizing the total number of flexible adjustments to the aligned structures. In contrast, MATT allows flexible adjustments to be made across the entirety of the aligned structures, even if such changes require structurally impossible bond angles or chain breaks, which are resolved at the end of the alignment procedure.

Different alignment methods use different measures of overall structural similarity and alignment significance. All four methods considered here produce a "raw" similarity score as well as a transformed "probability" score which attempts to account for the statistical significance of an alignment, given the length of the compared structures as well as that of alignment itself. Though in general both raw and probability scores are unique to the alignment method, there are some commonalities. CE, FATCAT and MATT all produce raw scores by summing a measure of AFP compatibility across the alignment path. In each of these three cases, the score function for individual AFP is a non-linear function of root-mean-square deviation (RMSD) of selected C_α_-C_α _associations between aligned structures. FATCAT and MATT also incorporate the number and degree of necessary flexible adjustments. Dali makes alignments using interatomic distance matrices rather than atomic coordinates directly. It thus uses a completely different similarity function, which is a parameterized, non-linear function of component submatrices selected by Monte Carlo search. As with raw scores, the probability scores used by these aligners share certain sets of traits. CE and FATCAT both compute probabilities using the empirical fit of a large set of scores to specific probability distributions, the Gaussian and Gumbel-type extreme-value distributions respectively. Dali computes a so-called Z-score by dividing raw scores by the output of a polynomial of degree four fit to the dependence of scores on alignment length. Finally, MATT transforms alignment scores using a non-linear function of the C_α_-C_α _RMSD of the alignment, the length of the alignment and the lengths of the aligned pair. The scoring functions of the four alignment methods are summarized in Table 1.

**Table 1:** Alignment scoring methods.

Method	Raw score	Probability score	Accounts for	Number of free parameters
CE	Sum of non-linear AFP scores	Fits scores to Gaussian distribution	Statistical significance	1 (μ = 0)

Dali	Non-linear matrix similarity function	Division by polynomial fit to length dependence of alignment score [21]	Length	4

FATCAT	Sum of non-linear AFP scores	Fits scores to extreme-value distribution [22]	Statistical significance	2 (ζ = 0)

MATT	Sum of non-linear AFP scores	Non-linear function of RMSD, chain and alignment lengths [11]	Statistical significance, length	3

Summary of key factors associated with the scoring strategies of CE, DALI, FATCAT and MATT. The rightmost column gives the number of free parameters in the probability scoring function or assumed probability distribution, not including raw score or alignment length. Parameters are considered "free" if they have to be estimated empirically or evaluated using a large set of structure alignment scores as a reference.

Except for the MATT probability score, all of the score functions in Table 1 produce similarity scores which must be converted to *dissimilarity *scores in order to be treated as distances. The transformation from similarity score to distance value is performed by subtracting the similarity scores from a maximum value. To prevent outlier distances from dominating, Equation (1) is used to select the maximum score *s_n _*as the value with the *p*^th ^percentile rank, by finding the index *n *of the *p*^th ^percentile of the N(N-1)/2 sorted similarity scores obtained between *N *structures. Throughout the following we set *p = 99.95%*.

Given pairwise similarity scores *s_i, j_*, for all pairs of structures *i *and *j*, the distances *δ_i, j _*are given by Equation (2).

(1) $$n=\frac{p}{100\times \left(\left(N\left(N-1\right)\right)/2+1\right)}$$

(2) $$\delta_{\text{i},\text{j}}=\left\{\begin{array}{c} {\text{s}}_{\text{n}}-{\text{s}}_{\text{i},\text{j}},\;{\text{s}}_{\text{n}}>{\text{s}}_{\text{i},\text{j}}|,i\neq j\\ 0\;,\;\;i=j\\ {\text{s}}_{\text{n}}\;,\;\;{\text{s}}_{\text{n}}\leq {\text{s}}_{\text{i},\text{j}} \end{array}\right)$$

The impact on PSS representations of each alignment method, as well as the various probability score transformations are described in the Results section.

### Obtaining low-dimensional maps of the structure space

MPSS of dimensionality *r *are created from a pairwise distance matrix ***δ ***using two algorithms for MDS. Classical MDS (CMDS) [23] produces a configuration of coordinates ***X ***which corresponds to a rank-*r *approximation of the experimental distance matrix, which is optimal in a linear least-squares sense. The classical MDS solution is obtained by converting ***δ ***to an inner product matrix ***A ***and recovering ***X ***via eigendecomposition. The relationship between the distances *d_ij_(**X**) *between points *i *and *j *in a putative configuration ***X ***and their inner product *A_ij _*is given by Equations (3) and (4). Note that ***A ***may be obtained directly from distance values because the diagonal summation terms found in both equations are equivalent to the combined row, column and grand means of the distance matrix.

(3) $$d_{ij}^{2}\left(X\right)=\sum_{k=1}^{r}X_{ik}^{2}+\sum_{k=1}^{r}X_{jk}^{2}-2\sum_{k=1}^{r}X_{ik}X_{jk}$$

(4) $$A_{ij}={\left(XX^{T}\right)}_{ij}=\sum_{k=1}^{r}X_{ik}X_{jk}=-\frac{1}{2}\left(d_{ij}^{2}\left(X\right)-\sum_{k=1}^{r}X_{ik}^{2}-\sum_{k=1}^{r}X_{jk}^{2}\right)$$

Equations (5) and (6) demonstrate the equivalence between some ***X ***generating ***A ***and the eigendecomposition of ***A ***(recall that the transpose of an orthogonal matrix is equal to its inverse).

(5) $$A=XX^{T}=\left(\lambda^{1/2}v\right){\left(\lambda^{1/2}v\right)}^{T}=v\lambda v^{T}=v\lambda v^{-1}$$

(6) Av=λv

Thus the *k*^th ^coordinate of the *i*^th ^point is found by multiplying the eigenvector *ν_ik _*of ***A ***by the square root of the corresponding eigenvalue *λ_k_*, as per Equation (7). Inner products of these coordinates form a rank-*r *approximation of ***A***.

(7) $$X_{ik}=\sqrt{\lambda_{k}}v_{ik},\;k\in \left[1,r\right]$$

Although the point configuration found by CMDS is optimal in a linear least-squares sense, CMDS does not explicitly minimize the stress induced between the MPSS distances and original pairwise distances, defined by Equation (8).

(8) $$\sigma \left(X\right)=\underset{i<j\leq N}{\sum }{\left(d_{ij}\left(X\right)-\delta_{ij}\right)}^{2}$$

As a consequence, the correspondence between pairwise and MPSS distances can be improved by perturbing an initial set of coordinates ***X***. Non-linear minimization of Equation (8) with respect to ***X ***is performed using a technique of convex analysis known as iterative majorization. In the context of MDS, this approach leads to an algorithm called scaling by majorizing a complex function (SMACOF) [24]. Briefly, SMACOF relies on the fact that stress can be monotonically decreased by iteratively perturbing ***X ***so as to minimize a quadratic ansatz representing a convex relaxation of Equation (8), proven in [24]. The solution to these successive convex relaxations reduces to the simple update function of Equation (9). Optimization of initial coordinates obtained using CMDS is achieved by repeated application of Equation (9) until the relative change in stress for an additional iteration falls below 10^-5^.

(9) $${x_{i}}^{\prime }=\frac{1}{N}\overset{N}{\underset{j}{\sum }}\left(x_{j}+\frac{\delta_{ij}\left(x_{i}-\left(x_{j}\right)\right)}{∥x_{i}-x_{j}∥}\right)$$

**Figure 1:**
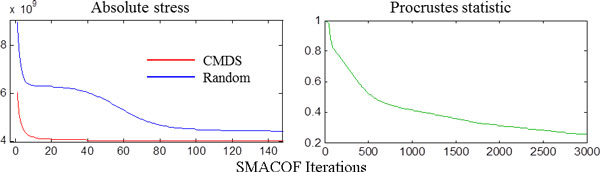
**Convergence of SMACOF with random and CMDS initializations**. Left: initial stress is much greater with the random initialization and takes many more iterations of SMACOF to converge. Right: The Procrustes statistic between the random and CMDS initializations shows that their SMACOF coordinates become very similar as the number of iterations increase.

Through the process described in Equation (9), the updated value ${x_{i}}^{\prime }$ of the *r*-dimensional *i^th ^*point $x_{i}$ is based on the values of the data points $x_{j}$. Conceptually, SMACOF minimizes stress by attempting to "fold" the unaccounted for higher dimensional separations into the *r *dimensions of the point cloud. During this process, the position of each point is influenced by all of the others, leading eventually to a configuration of coordinates which is maximally consistent with the pairwise distances given the constraints of the chosen dimensionality. Even though SMACOF computations are initialized with the results of classical scaling, SMACOF should not be seen as merely refining the CMDS coordinates. SMACOF will converge eventually given any particular starting condition. However, the CMDS configuration provides a high quality initial guess which contributes to rapid convergence. This is demonstrated by Figure 1, which shows the absolute stress of a configuration versus the number of completed SMACOF iterations for both uniformly random and CMDS initializations. The figure also plots the Procrustes statistic describing the fit between the two sets of coordinates after an optimal linear transformation including translation, rotation and scaling has been applied. As seen in the figure, the match between the initialization conditions improves as the number of complete SMACOF iterations increases, both in terms of the stress induced by the configurations and the difference between them. While many repeated iterations are required in order for the two MPSS to converge, the initialization with CMDS allows minimum stress to be reached extremely rapidly.

**Figure 2:**
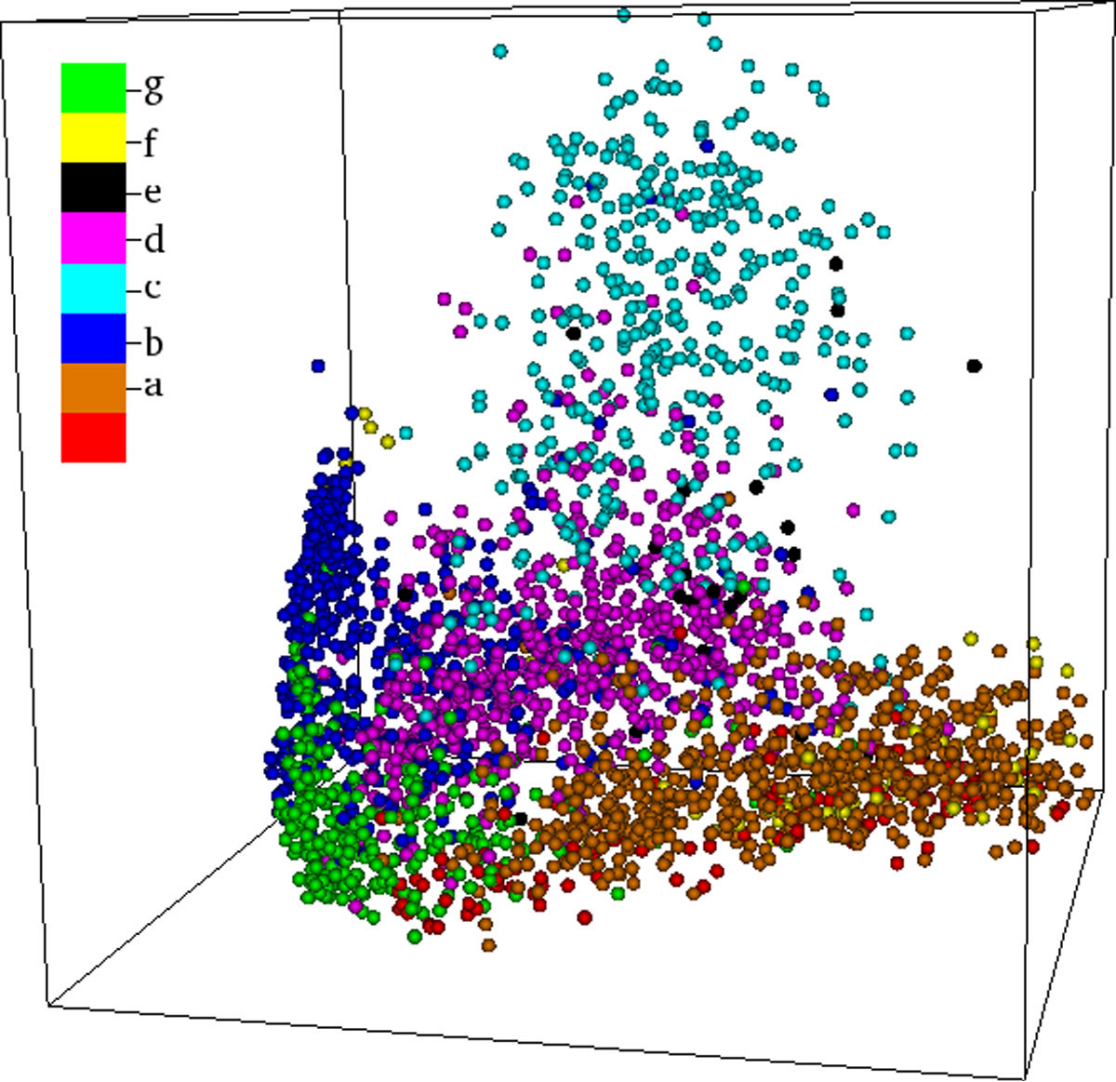
**MPSS MATT(C3)**. A 3D MPSS, constructed using CMDS in conjuction with raw MATT distances. Points in the MPSS are colored by SCOP Class. The reader may note the strong separation of the major protein classes. In particular, small proteins ('g,' green) cluster densely near the origin, while the all alpha ('a,' brown) and all beta ('b,' blue) classes form two roughly orthogonal axial structures. Between these lies the α+β class ('d,' magenta), with the α/β class ('c,' cyan) rising high above the α,β plane.

**Figure 3:**
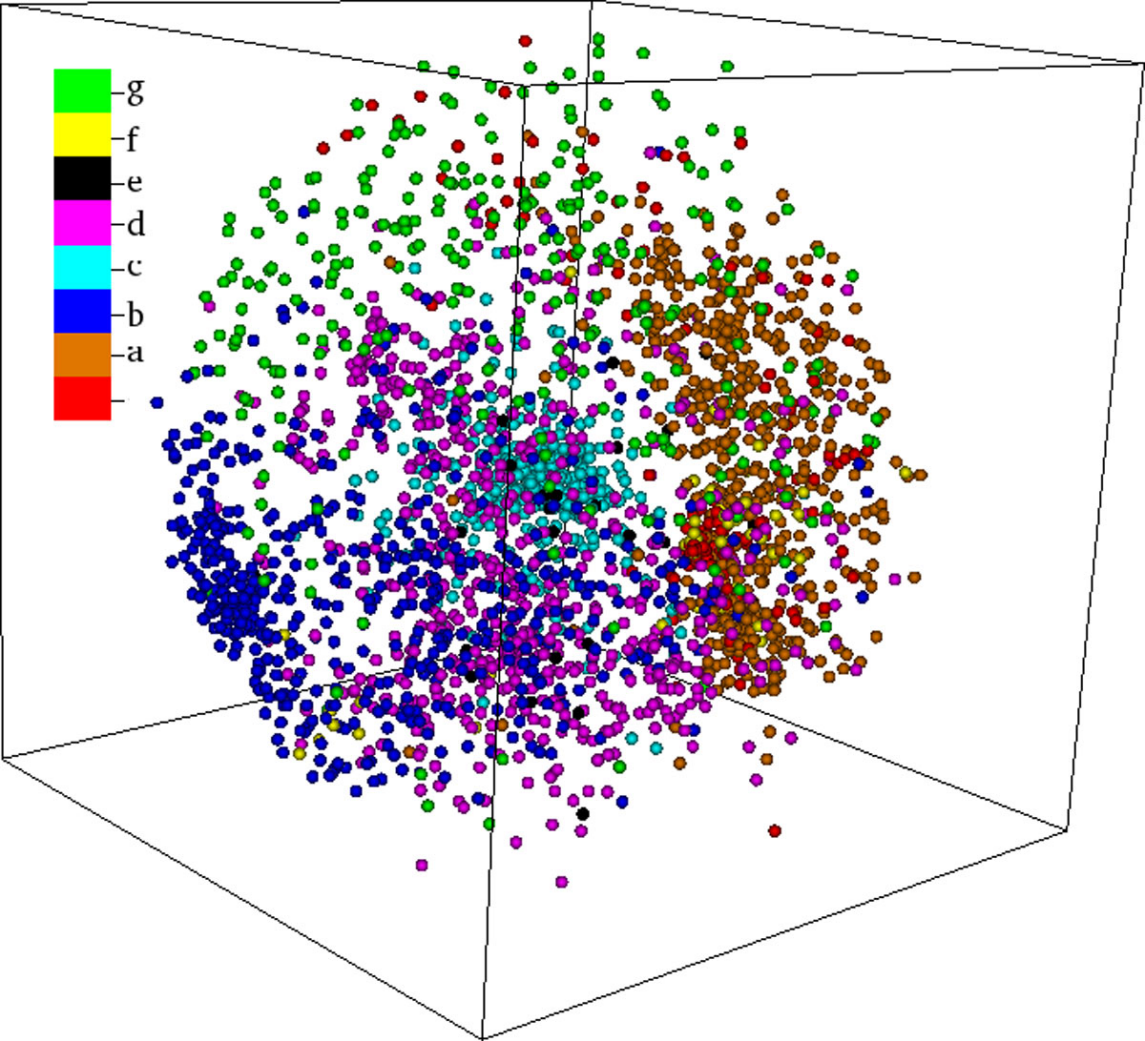
**MPSS MATT(S3)**. A 3D MPSS constructed using SMACOF and raw MATT distances. As in Figure 2, the protein classes are well separated. However, the qualitative appearance of the MPSS is very different and points in the MPSS appear to lie near a spherical manifold. Compared with Figure 2, the α/β class ('c,' cyan) is very tightly clustered, while the small proteins ('g,' green) are spread farther apart.

Figures 2 and 3 present examples of three dimensional MPSS, which have been proposed and used by us as tools for holistic and interactive visualization, exploration, and sensemaking of the structure space [14]. These figures depict the first MPSS to be created using raw MATT distances with both CMDS (Figure 2) and SMACOF (Figure 3). The proteins in the MPSS are colored by their SCOP Class, in order to convey the high interpretability of the maps.

### Classification prediction and receiver operating characteristics

**Figure 4:**
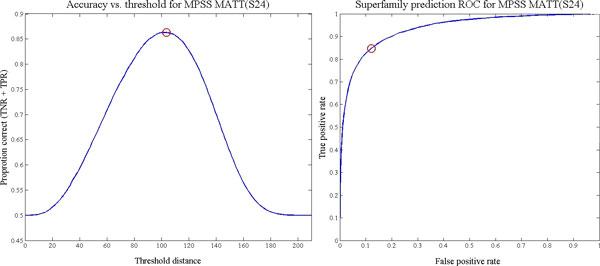
**Example ROC and threshold-accuracy curves**. A) Plot of true classification rate vs. threshold distance for a 24D MPSS based on Dali and SMACOF. The maximum, corresponding to the intersection of the ROC curve and the iso-performance tangent, is marked in red. B) ROC curve for the same set of MPSS distances. The point associated with the best threshold from A) is indicated by the red circle.

A PSS representation can be used to predict the functional relationships described by a set of annotations which are taken as a gold standard. In this work, such a standard is provided by the remotely homologous Superfamily and Fold levels of the hierarchical SCOP database. A binary classifier which separates distances that indicate a functional relationship from those that do not can be constructed by selecting a threshold distance representing the size of a neighborhood in structure space. For a particular neighborhood size, the number of true and false negatives and positives can be calculated by comparing the adjacency matrix of the standard to PSS adjacencies obtained by thresholding the PSS distances. That is, a binary matrix is computed by thresholding each element of a distance matrix, and each element is checked against the corresponding element of a distinct binary matrix derived from the known relationships in SCOP. Varying the neighborhood size parameter between a minimum of zero and the maximum distance in the data allows construction of a complete receiver operating characteristic (ROC) curve, which represents the overall accuracy of annotation inference in terms of the tradeoff between the false positive rate (FPR) and true positive rate (TPR). This accuracy can be expressed as the area under the ROC curve (AUC), which takes values in the interval [0, 1]. We calculate the AUC by numerical integration of the ROC curve using the trapezoidal rule. The best single threshold for binary classification of the intermolecular distances can be found by selecting the threshold which maximizes the total true classification rate (TCR), or sum of TPR and true negative rate (TNR) [25]. Example TCR and ROC curves, computed for a 24D SMACOF MPSS based on raw MATT distances, is shown in Figure 4. This TCR curve exemplifies the ease of locating such maxima. Note that unlike [11] we compute classification statistics exhaustively rather than using the approximate sampling algorithm proposed in [25].

### Hierarchical distance clustering

We use the hierarchical clustering method first presented in [11] for clustering data in MPSS. This method seeks to reproduce a given level of SCOP with no parameterization or supervision except for specification of the optimal thresholds learned during ROC analysis. Similar to other methods for hierarchical clustering, data points are first linked to form a dendrogram. The tree is built using strict neighbor joining (NJ) as implemented in the ClearCut program [26]. Next, the input space is tessellated based on 1) the topology of the cluster tree and 2) a criterion for separation or merging of the data points (leaf nodes) into individual clusters. Specifically, clusters are formed by recursively descending the tree, forming a cluster whenever the distances between all descendent leaves of a particular subtree fall within the threshold (maximum-linkage clustering). The topology of the dendrogram restricts potential cluster members, which allows the single threshold to accurately recreate clusters of different densities, so long as the density of a cluster is not so low that the points do not fall within the threshold distance (and inasmuch as the hierarchy is correct).

**Figure 5:**
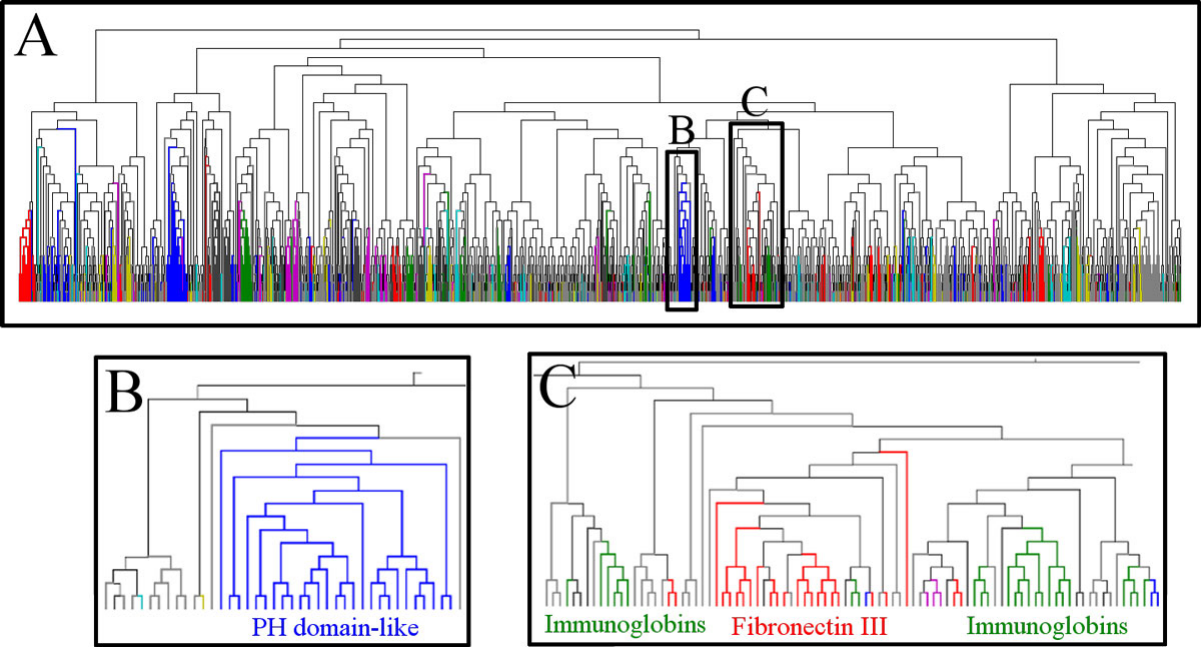
**Neighbor-joining tree for Dali(S12)**. A) The complete cluster tree for distances from the 12D SMACOF MPSS based on Dali. Colors indicate membership in the 200 most populated SCOP Superfamilies. Because it is difficult to distinguish highly similar colors, a smaller number of distinct colors are reused periodically across the tree, while guaranteeing that color labels are locally unique. The reader may note that different superfamilies are found at different depths within the tree, so that the use of a single threshold for all clusters is only approximate. B) The part of the tree that corresponds to the PH domain-like superfamily. This superfamily is entirely and exclusively contained within a single subtree. C) The region of the tree containing the Immunoglobin and Fibronectin Type III superfamilies. These superfamilies do not correspond either to a single subtree or to a single depth within the tree.

Figure 5 shows an example NJ cluster tree, computed using distances from the 12D Dali MPSS computed with SMACOF. The relationship between the empirical cluster tree and SCOP hierarchy is visualized by coloring nodes belonging to the 200 most populated superfamilies. Due to the difficulty in distinguishing a large number of similar colors, we periodically reuse colors while drawing the tree, but in such a way that all color labels are locally unique. The layout of the colored tree is generated using ColorTree [27] and drawn with Dendroscope [28]. This figure allows several conclusions regarding the hierarchical clustering procedure to be drawn. First, while most superfamilies are found at broadly similar tree depths, there are a significant number of superfamilies which branch at relatively shallow levels of the tree (Figure 5A). As alluded to above, these structurally diverse superfamilies are unlikely to be well represented when using a single threshold. Second, it is clear that many superfamilies correspond closely (if not exactly) with a single subtree, such as the PH domain-like superfamily isolated in Figure 5B. However, there are also many superfamilies which are not captured successfully by the NJ cluster tree, such as the Immonoglobin and Fibronectin III superfamilies depicted in Figure 5C. Such groups are not likely to be accurately reproduced by hierarchical clustering, even though the distances between member nodes may be within the threshold. Despite the limitations of maximum-linkage hierarchical clustering (with a single threshold), most clades are well captured, and it provides a highly useful method for comparative analysis of PSS representations as explored further in the Results section.

## Results and discussion

### Experimental approach

We aim to exhaustively compare all PSS representations that can be constructed based on the collection of alignment, score transformation and low-dimensional projection methods described above. A straightforward way to evaluate the power of a PSS representation for classification of protein structures is to compare distances between neighboring points against gold standard annotations of functionally and structurally similar proteins. Here we use two sets of annotations, the remotely homologous Superfamily and Fold levels of the SCOP classification hierarchy, as standards. The ROC framework is then used to analyze prediction of shared annotation for protein pairs. The results demonstrate the efficacy of MPSS distances for automatic structure classification, and are corroborated by examples showing the mapping of selected SCOP Superfamilies. ROC analysis is also used to study in detail the impact of alignment score transformations on the representation of PSS, as well as the dimensionality dependence of MPSS-based classification. Finally, we use maximum-linkage hierarchical clustering to tessellate PSS representations and analyze the similarity of the result to the clusters specified by SCOP.

Due to the large number of PSS representations studied, we refer to them using a simple code. The code contains the alignment algorithm name, and in parenthesis the score type, MDS method and dimensionality. A capital "Z" denotes probability scores (raw scores are considered the default, so no letter is used), while a capital "C" denotes the use of classical scaling and "S" denotes SMACOF. Finally, if a specific MPSS dimensionality is intended, the dimensionality is given as a number. Following these conventions, "Dali" refers to raw Dali alignment distances, while "Dali(Z)" refers to the probability scores. "Dali(C)" and "Dali(ZC)" refer to MPSS (of all dimensionalities) using classical scaling and Dali raw or probability scores, respectively. As a final example, "Dali(S12)" refers specifically to the Dali raw score/SMACOF MPSS of dimension 12.

### Classification performance

We computed ROC curves for prediction of SCOP Superfamily and Fold membership using all 8 sets of alignment distances and all 16 types of MPSS distances. For each MPSS distance, ROC curves were generated using MPSS of several dimensionalities ranging from 3 to 120. The overall accuracy of annotation prediction is expressed using the ROC AUC, which equally values maximization of true positives and minimization of false ones (sensitivity and specificity). Table 2 and Table 3 give the AUC, for Superfamily and Fold prediction respectively, of the best-performing score type and MPSS drawn from all four alignment methods. The best overall performance - considering both Fold and Superfamily levels - is obtained with the 12D MPSS constructed from raw Dali distances using SMACOF, with Superfamily and Fold AUC of 93.85% and 90.42%, respectively (average 92.14%).

**Table 2:** Top performing PSS representations for SCOP Superfamilies.

Best alignment score	AUC	Best MPSS	AUC
**CE(Z)**	91.25%	**CE(ZS24)**	91.96%
**Dali(Z)**	87.43%	**Dali(ZS30)**	***94.15%***
**FATCAT(Z)**	91.16%	**FATCAT(C60)**	90.54%
**MATT(Z)**	92.02%	**MATT(S24)**	93.85%

The AUC of the best score type of each aligner, as well as of the best MPSS, are shown. The best AUC in the table (for the Dali MPSS) is highlighted in bold italics. Note that a particular MPSS always improves upon the underlying alignment algorithm and score transformation.

**Table 3:** Top performing PSS representations for SCOP Folds.

Best alignment score	AUC	Best MPSS	AUC
**CE(Z)**	86.89%	**CE(ZS12)**	89.23%
**Dali(Z)**	78.67%	**Dali(S12)**	***90.42%***
**FATCAT(Z)**	85.51%	**FATCAT(C6)**	87.80%
**MATT(Z)**	85.29%	**MATT(ZC24)**	89.78%

The AUCs for SCOP Folds. The reader may note that in this case also, the MPSS improves upon the underlying alignment algorithm and score transformation.

### Study of selected SCOP superfamilies using MPSS

**Figure 6:**
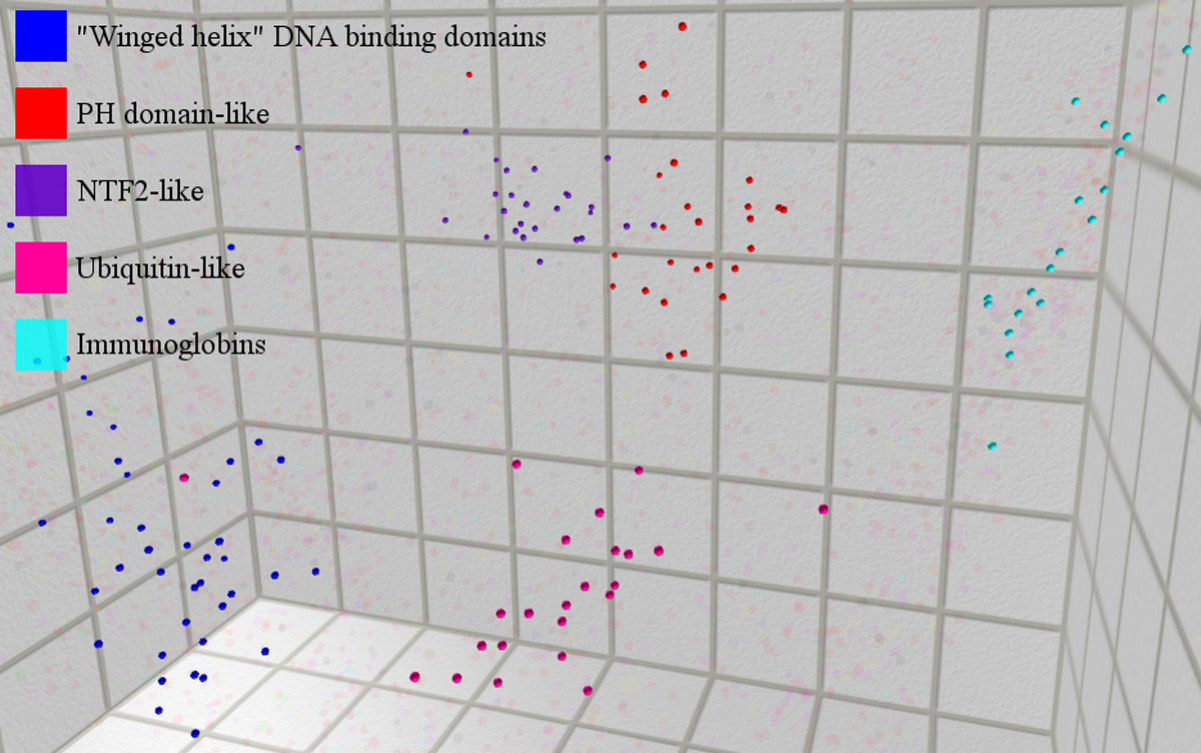
**MPSS with selected SCOP superfamilies highlighted**. The figure shows a view of FATCAT(C3), with several selected SCOP superfamilies highlighted. Points representing members of other superfamilies are transparent and dimmed, but present in the map. It can be seen that the superfamilies are well separated in the mapping space.

The ability of MPSS distances to predict the classifications of remotely homologous protein structures can be clearly illustrated by visualizing maps of suitably low dimensionality and examining the distributions of points corresponding to individual superfamilies. Due to the large size of the space as well as the presence of more than 1,000 superfamilies, we have focused on a region of the PSS containing several of the most highly populated SCOP Superfamilies present in our data. Figure 6 depicts this region in a three-dimensional MPSS, specifically FATCAT(C3). Proteins belonging to each of these superfamilies are highlighted in color, while the other points in the space have been hidden. Based on Figure 6, it is apparent that there are variations in the shape, density and average positions of the different superfamily distributions. The Ubiquitin-like and Immunoglobin superfamilies, for example, are relatively cohesive and apart from other groups, while the dense NTF2-like and PH domain-like clades are immediately adjacent to one another. The "Winged helix" DNA binding domains are relatively less dense when compared to the other groups shown. This observation is indicative of a higher level of structural diversity within this clade.

Despite the marked, qualitative variations between the superfamilies, they are all well captured by the optimal distance threshold determined using the ROC analysis. The results of classification prediction within these specific superfamilies, using the optimal threshold, can be summarized using precision, recall and their harmonic mean (F_1_-measure). These statistics are given for each of the selected superfamilies in Table 4. All of these superfamilies are accurately captured using the ROC distance threshold, and the lowest F_1 _score is 0.866. However, the reader may note that the widely dispersed "Winged helix" superfamily is predicted with a somewhat lower recall than are the other superfamilies. This is a consequence of the use of a single threshold for all groups; a threshold which performs admirably for relatively homogenous groups such as the NTF2-like superfamily may be too restrictive to capture all of the members of a diverse group such as "Winged helix" domains.

**Table 4:** Classification performance within selected SCOP superfamilies.

Superfamily	"Winged-helix"	PH domain-like	NTF2-like	Ubiquitin-like	Immunoglobin
**Precision**	0.997	1	1	1	0.987
**Recall**	0.766	0.818	0.982	0.909	0.811
**F_1_-measure**	0.866	0.900	0.991	0.953	0.890

Classification performance statistics are given for each of the five superfamilies shown in Figure 6. Prediction of SCOP superfamily membership is made by thresholding MPSS distances from FATCAT(C3) with the (single) optimum threshold obtained from ROC analysis. Statistics presented in the table include: precision or positive predictive value, recall or true positive rate, and the F_1_-measure, which is the harmonic mean of precision and recall.

### Score transformations and performance

In order to succinctly present the impact of score transformation on classification using raw and MPSS distances alike, we compute the ratios between the AUC obtained by the top-performing MPSS, and the transformed and untransformed alignment distances. These are shown in Table 5, for SCOP Superfamily, and in Table 6 for SCOP Fold.

**Table 5:** Relative performance of MPSS and PW distances for SCOP Superfamilies.

	CE	CE(Z)	Dali	Dali(Z)	FATCAT	FATCAT(Z)	MATT	MATT(Z)
Z : Raw	**1.500**		**1.003**		**1.083**		**1.037**	
CMDS : PW	**1.415**	1.000	0.893	0.894	**1.075**	0.963	**1.041**	0.998
SMACOF : PW	**1.359**	**1.008**	**1.079**	**1.077**	**1.037**	0.912	**1.057**	0.998
SMACOF : CMDS	0.960	**1.008**	**1.207**	**1.204**	0.965	0.948	**1.015**	1.000

The relative performance for Superfamily prediction of both raw and probability (Z) scores are given for each aligner, as are the relative performances of MPSS of both types (CMDS and SMACOF) versus pairwise distances (PW) as well as each other. Relative performance is expressed as ratios of AUC values, with ratios greater than one presented in bold. In each case, the MPSS with the highest AUC is used.

**Table 6:** Relative performance of MPSS and PW distances for SCOP Folds.

	CE	CE(Z)	Dali	Dali(Z)	FATCAT	FATCAT(Z)	MATT	MATT(Z)
Z : Raw	**1.588**		**1.002**		**1.137**		**1.063**	
CMDS : PW	**1.525**	0.993	0.964	0.918	**1.167**	0.993	**1.114**	**1.053**
SMACOF : PW	**1.418**	**1.027**	**1.152**	**1.137**	**1.088**	0.937	**1.106**	**1.043**
SMACOF : CMDS	0.929	**1.034**	**1.195**	**1.240**	0.932	0.943	0.993	0.991

Fold classification performance. The convention used for presenting the data is the same as in Table 5.

For prediction of membership in SCOP Superfamilies and Folds alike, all of the score transformations provide an improvement over raw pairwise distances. This improvement ranges from over 50% for CE to nearly nothing for Dali. MPSS likewise improve performance over pairwise distances, but do not consistently improve the performance after score transformations are already applied. For Superfamilies, the MPSS computed using SMACOF tend to outperform those based on CMDS, but the opposite holds at the Fold level. The specific results for particular structure aligners, score transformations and MDS algorithm are discussed in detail below.

The results presented in Table 5 and Table 6 lead to certain conclusions about the different score transformations. CE uses the simplest transformation, which converts scores to probabilities by assuming a Gaussian distribution. Although the Gaussian assumption may be questionable, the central limit theorem provides that many distributions approximate a Gaussian for sufficiently large sample sizes. Furthermore, while a Gaussian fit technically requires two free parameters, subtraction of the sample mean renders the population mean basically irrelevant and overfitting rather unlikely. This transformation is the only one which provides an unequivocal benefit for both pairwise distances and MPSS.

In contrast, the transformation used by Dali accounts only for the relationship between alignment score and length of the aligned structures, as opposed to statistical significance. Based on Table 5 and Table 6, it appears this model has little impact on classification of remotely homologous structures of shared function. It has been argued previously that the down-weighting of alignments between large structures impairs the ability of Dali Z-scores to capture functional relationships implied by local similarity between such structures [12]. However, we find only that these Z-scores offer little improvement. With regard to MPSS, the transformation has essentially no effect at the Superfamily level and a somewhat negative effect at the Fold level.

FATCAT takes an approach to probability transformation which is akin to that of CE - probabilities are calculated by fitting scores to a chosen distribution. Rather than a simple Gaussian, FATCAT employs the extreme value distribution, which is fit numerically via a simplex search across three free parameters. Use of this method leads to a significant improvement over raw FATCAT alignment distances, but impairs the efficacy of MPSS.

The probability transformation used by MATT is distinct from the other pairwise score types in that it already represents a dissimilarity or distance rather than a similarity score. The transformation itself is a non-linear function of the final Cα-Cα RMSD of the aligned residues, the lengths of the aligned structures, the length of the alignment and three empirically determined parameters. These parameters were set by finding the slope of a line which best discriminates between real and decoy alignments on a graph of RMSD versus alignment length. While this transformation has a large, positive effect for pairwise distances, it too impairs the efficacy of MPSS.

We find that FATCAT and MATT Z-score transformations reduce the performance of MPSS by introducing and/or exacerbating violations of the triangle inequality in the pairwise distances, detectable as negative eigenvalues in the inner product matrix ***A ***(data not shown). Such violations of course preclude accurate representation by points in Euclidean space. In contrast, the Dali transformation does not have such an effect and the CE transformation actually reduces degree of triangle inequality violations (fewer/smaller negative eigenvalues). Nevertheless, MPSS computed using raw scores provide the best overall performance.

Projection into an explicit coordinate representation provides a score transformation method which simultaneously outperforms extant probability transformations and alignment length adjustment functions but relies on only a single free parameter (MPSS dimensionality) and makes no assumptions about any underlying distribution of scores. Instead, individual proteins are localized in an explicit spatial representation which is mutually consistent with all pairwise distances simultaneously. In a MPSS, sets of proteins with many highly significant pairwise distances are found in distinct, potentially overlapping, regions far from those inhabited by proteins with which they bear no structural relationships. MPSS are not highly sensitive to the dimensionality parameter; the results presented here indicate that MPSS represent the protein space in a highly accurately with dimensionalities ranging from 12 to 30, and in many cases perform admirably with as few as three dimensions. Such MPSS are suitable for visual inspection and interpretation, a topic we have addressed elsewhere [14].

### Dimensionality and performance

**Figure 7:**
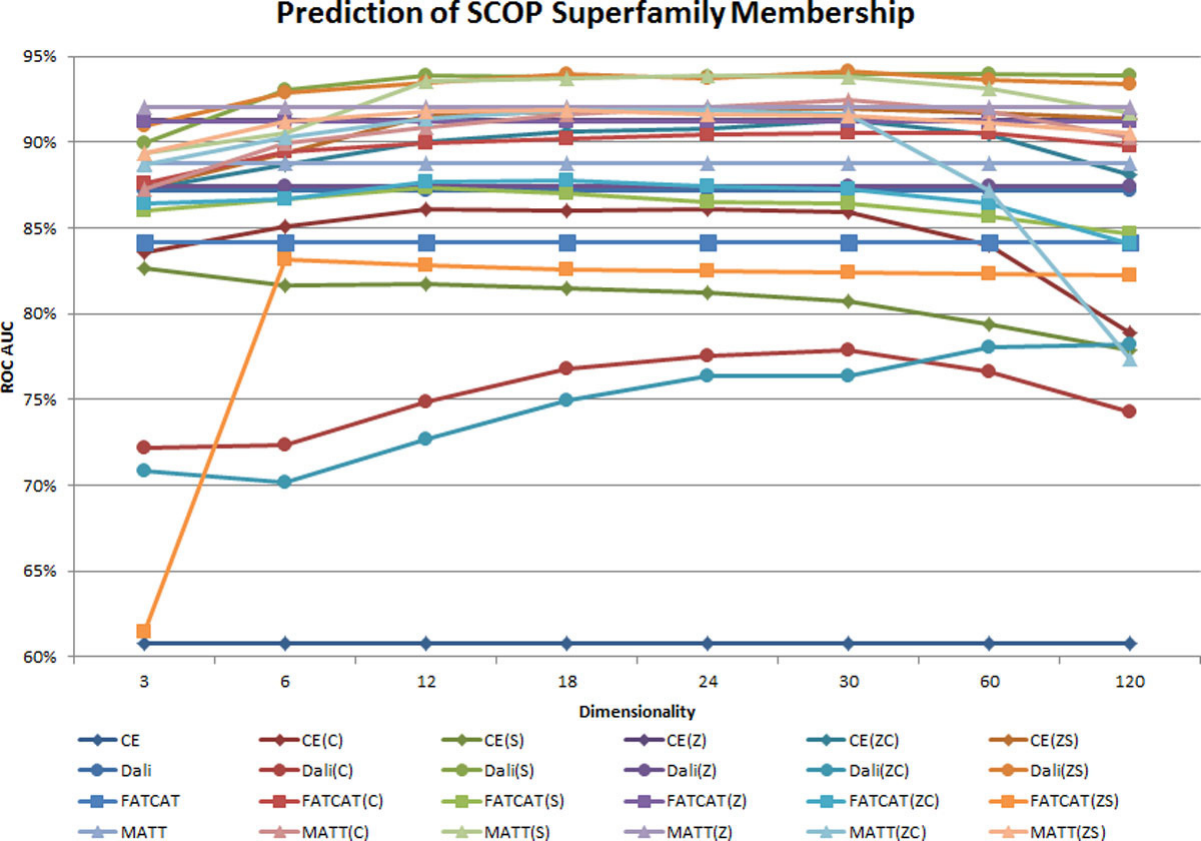
**AUC for SCOP Superfamily prediction vs. MPSS dimensionality**. The Superfamily classification AUC are shown for all 24 PSS representations. The AUC is plotted against MPSS dimensionality; pairwise distances are depicted as a flat line for reference. The legend gives the curves using the PSS name code containing the aligner name, Z for probability scores, C for classical scaling and S for SMACOF. Detailed descriptions of the trends found in this figure are given in the text. The figure shows that the MPSS Dali(S12-30) and MATT(S12-30) are most accurate.

**Figure 8:**
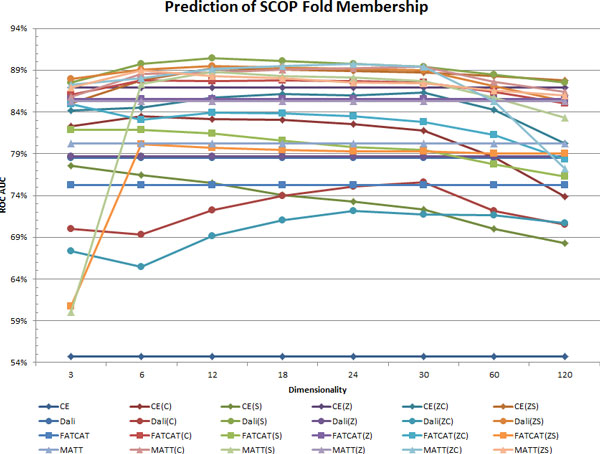
**AUC for SCOP Fold prediction vs. MPSS dimensionality**. Fold classification AUC are shown for all 24 PSS representations. The AUC is plotted against the MPSS dimensionality; pairwise distances are depicted as a flat line for reference. Detailed descriptions of the trends found in this figure are given in the text. The figure shows that the MPSS Dali(S12) is the most accurate at this SCOP level.

The AUC values for prediction of SCOP Superfamily and Fold memberships are plotted versus MPSS dimensionality in Figure 7 and 8, respectively. As mentioned previously, the tested dimensionalities range from 3 to 120. The AUCs obtained using the pairwise PSS representations are displayed as flat lines, so they may be referenced easily across each figure.

Several interesting trends are revealed by the dependence of MPSS distances on dimensionality. At the Superfamily level, Dali MPSS with only three dimensions already surpass pairwise distances in AUC, while for CE and MATT MPSS do not do so for dimensionalities less than 12. SMACOF outperforms CMDS at the Superfamily level, in general obtaining higher classification accuracies with lower dimensionalities. Two key differences are seen in the context of Fold prediction. First, MPSS distances generally perform better than pairwise distances even at the lowest dimensionalities considered in Figure 7 and 8. Second, MPSS at the Superfamily level maintain a plateau of performance for dimensionalities of 12 to 30, but at the Fold level the decline in performance with increasingly dimension is more rapid in general. The drop off in classification accuracy at a particular dimensionality for each aligner is to be expected because insomuch as MPSS perform better than pairwise distances, higher dimensionalities which permit precise replication the original distances will lead to worse performance. Although inflection points occur at the same dimensionalities, the AUC rises and falls more steeply than for SMACOF than for CMDS.

The dimensionality dependence of MPSS representations in general, as well as their superiority to pairwise distances can be understood based on the nature of the underlying algorithms used for low-dimensional projection. CMDS employs a linear (eigen-) decomposition into a set of mutually orthogonal axes (eigenvectors) which have the property of accounting for increasingly smaller proportions of the total dispersion between points (eigenvalues). The combination of these two restrictions forces random noise present in the distances to be distributed across many small linear components (eigenvectors with small eigenvalues). With respect to distances, the dimensionality reduction of CMDS thus acts as a noise-reducing filter. However, any non-linear correlation structure is may also be distributed amongst a large number of linear components and subsequently filtered out in the resulting MPSS distances, as may any highly local relations involving only a small number of structures. In contrast to CMDS, SMACOF non-linearly minimizes the deviation between pairwise and MPSS distances. The restricted dimensionality of the map acts as a smoothness constraint which prevents noise - which is assumed to be non-smooth - from influencing the MPSS. However, SMACOF may overfit large noise terms which would otherwise be ignored by CMDS for a given dimensionality. The right choice depends on the non-linearity of the relations under study (i.e. SCOP Fold and Superfamily relationships) as well as the noise magnitude of the pairwise distances. The steeper slopes seen for SMACOF in both Figure 7 and 8 occur because SMACOF effectively transfers higher dimensional information contained in pairwise distance data into the low-dimensional projections created by CMDS. At lower dimensions this translates into higher performance, as valuable information discarded by CMDS is introduced into the MPSS. The converse is observed at higher dimensionalities, because CMDS adds new dimensions in order of decreasing eigenvalue. When such MPSS are refined, the SMACOF algorithm is left to fit only the intermolecular separations explained by the smaller, unused eigenvalues. When these eigenvalues correspond primarily to noise, stress majorization leads to overfitting. These observations also help to explain the trends seen for the various aligners with respect to Z-score transformations, which carry explicit restrictions on higher-order statistical moments (e.g. CE, FATCAT), down-weight particular alignment lengths (Dali), or both (MATT). In contrast, MPSS generated with CMDS may represent structures which decompose into a linear basis, while SMACOF is limited only in terms of the geometries possible in Euclidean spaces of finite dimension.

### Alignment algorithms and sparsity

**Figure 9:**
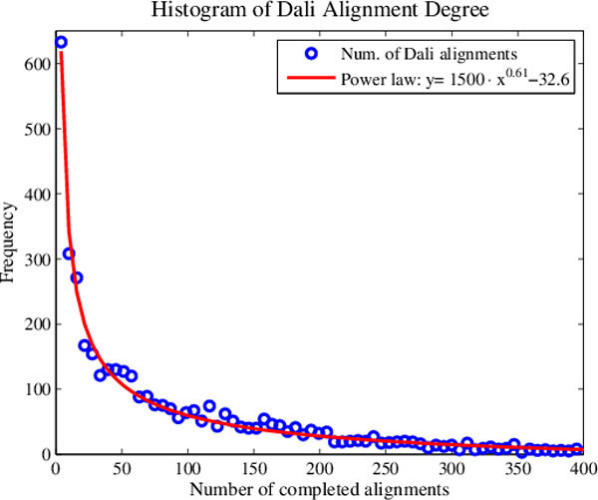
**Histogram of completed Dali alignments**. A 100-point histogram of the number of successful Dali alignments for each structure. The histogram counts fit tightly to a power law, *R^2 ^= 0.988*. This corroborates the hypothesis that high-degree nodes mediate the relationships between most other pairs of proteins in a scale-free manner.

In light of the results presented in the preceding sections, it is possible to make some statements regarding the individual alignment methods. The rigid alignments of CE appear to be characterized by a significant amount of noise, which can be filtered probabilistically or via CMDS. CMDS performs less well at the Fold level, but SMACOF maps using CE Z-scores are able to recover useful information discarded by CMDS without overfitting of noise. Dali makes for an interesting case because it limits the alignment search space by filtering out pairs deemed unlikely to align and is alone among the four aligners in frequently failing to perform particular alignments. Indeed, while CE, FATCAT and MATT aligned all possible pairs in our data set, Dali was able to calculate an alignment score for just 2% of approximately 8 million pairs. As a consequence of this extreme sparsity, Dali scores do not predict Superfamily or Fold membership particularly well. In a seeming paradox however, Dali-based MPSS constructed with SMACOF obtain better observed performance than all other representations studied. Most likely, the pre-alignment filter results in a low-noise data set but the high level of sparsity prevents pairwise comparisons from being informative for remotely homologous structures. SMACOF works well in this case because the algorithm is able to infer the correct relative distances of un-aligned pairs based on transitive relationships between the alignment distances which do exist. Specifically, these transitive relationships are mediated by a small number of frequently aligned structures in a scale-free fashion. This conclusion is strongly supported by the fact that a histogram of the number of partners aligned per structure is closely fit by a power law (*R^2 ^= 0.988*), as shown in Figure 9.

FATCAT is significantly aided by probability scoring using the extreme-value distribution and performs fairly well at the Superfamily level, but has middling performance for prediction of Fold. CMDS improves performance of FATCAT raw scores for Fold prediction, but SMACOF MPSS distances perform worse than pairwise distances, and at the Superfamily level FATCAT MPSS perform worse in general. These data suggest that FATCAT distances contain less noise than the other alignment distances, especially for alignments approximating Superfamily-type relationships, but that FATCAT is not as sensitive as the other algorithms to more distant homologies. Finally, MATT is the most recently published of the aligners and achieves the best Superfamily performance for pairwise distances. At this level, MATT SMACOF MPSS perform nearly as well as the top performing Dali MPSS. Like FATCAT, MATT is highly sensitive to the homologies found within Superfamilies, but is not as sensitive to the more distant relationships found at the Fold level. That the 24D MPSS created with CMDS and MATT raw scores is second only to the robust combination of Dali and SMACOF for prediction of Fold membership suggests that CMDS effectively discards Fold-level noise present in the MATT distances.

### Hierarchical clustering

ROC AUC values evaluate representations of PSS in terms of their ability for colocalization of related pairs of protein structures, but do not say whether or not a tessellation of PSS can naturally reproduce an arrangement of mutually exclusive clusters such as that given by the SCOP classifications of a given level. To investigate how closely clusters within different PSS representations are able to replicate the clades found at a given level of the SCOP hierarchy, we use maximum-linkage hierarchical clustering, following the approach taken in [11]. First, the classification given by SCOP and the results of automatic clustering are compared by finding the SCOP group most similar to each cluster. Then, the relative similarities of these sets of protein structures are investigated, as well as the degree to which members of a single group in one tessellation may be divided amongst several groups in the other. The results of cluster analysis for SCOP Superfamiles are listed in Table 7 and those for the Fold-level classification in Table 8. The analysis is bidirectional; thus each table separately describes the mapping from hierarchical clusters to SCOP clades as well as the inverse mapping from SCOP clades to particular hierarchical clusters.

**Table 7:** Comparison of SCOP superfamilies and hierarchical clusters.

Cluster mapping	**Max deg**.	**Mean deg**.	**Std deg**.	**Mean sim**.	**Std sim**.	Mapped clusters	Ratio-1
**SCOP- Matt(Z)**	171	***1.928***	6.927	***0.394***	0.309	***1180 : 612***	***0.928***

**SCOP-Matt(S12)**	***22***	2.453	***2.324***	0.285	0.224	1180 : 481	1.453

**SCOP-Dali(S12)**	51	2.987	3.920	0.249	***0.219***	1180 : 395	1.987

**SCOP-Matt(S24)**	29	2.370	3.121	0.312	0.268	1180 : 498	1.369

**SCOP-Dali(S30)**	52	2.582	3.613	0.294	0.255	1180 : 457	1.582

**Matt(Z)- SCOP**	35	1.262	1.597	***0.492***	0.319	766 : 607	0.262

**Matt(S12)-SCOP**	22	1.180	1.074	0.400	0.251	564 : 478	0.180

**Dali(S12)-SCOP**	***9***	***1.082***	***0.521***	0.419	***0.247***	***422 : 390***	***0.082***

**Matt(S24)-SCOP**	19	1.178	0.942	0.464	0.280	590 : 501	0.178

**Dali(S30)-SCOP**	20	1.101	0.944	0.467	0.272	502 : 456	0.101

The best results for each mapping direction and column are highlighted in bold italics. Max, mean and std. degree refer to the number of Superfamilies or clusters corresponding to a given superfamily or group. Mean and std. similarity refer to the Jaccard coefficients obtained between these matching clusters. Finally, "mapped clusters" indicates the number of clusters or Superfamilies found, and the number of unique groups which to which are mapped. This ratio minus one is shown in the rightmost column; the closer this number is to zero, the more similar are aggregate sizes of the two tessellations. The values of min degree and max similarity are both one and the value of min similarity is zero.

**Table 8:** Comparison of SCOP folds and hierarchical clusters.

Cluster mapping	**Max deg**.	**Mean deg**.	**Std deg**.	**Mean sim**.	**Std sim**.	Mapped clusters	Ratio-1
**SCOP- Matt(Z)**	98	***1.554***	4.432	***0.419***	0.311	***757 : 487***	***0.554***

**SCOP-Matt(S12)**	***10***	1.966	***1.391***	0.294	0.222	757 : 385	0.966

**SCOP-Dali(S12)**	25	2.419	2.245	0.257	***0.204***	757 : 313	1.419

**SCOP-Matt(S24)**	17	2.00	1.974	0.309	0.248	757 : 379	0.997

**SCOP-Dali(S30)**	22	2.132	2.001	0.310	0.249	757 : 355	1.132

**Matt(Z)- SCOP**	37	1.552	2.323	0.391	0.321	762 : 491	0.552

**Matt(S12)-SCOP**	15	1.296	1.127	0.346	0.244	495 : 382	0.296

**Dali(S12)-SCOP**	***7***	***1.150***	***0.518***	0.365	***0.231***	***361 : 314***	***0.150***

**Matt(S24)-SCOP**	20	1.347	1.448	0.367	0.268	516 : 383	0.347

**Dali(S30)-SCOP**	10	***1.150***	0.643	***0.426***	0.266	***413 : 359***	***0.150***

The best results for each mapping direction and column are highlighted in bold italics. Column headings are the same as in Table 7, but for SCOP Folds rather than for Superfamilies. The values of Min degree and max similarity are both one and the value of min similarity is zero.

The comparison between a cluster and its most similar SCOP group (and vice-versa) is made in terms of descriptive statistics detailing the closeness of the map, and descriptive statistics are compiled across all such pairings. These statistics include the mean, standard deviation and maximum degrees to which a SCOP clade is divided across multiple clusters (or vice-versa), as well as the mean and standard deviation of the Jaccard coefficient between them. The Jaccard coefficient is an index of the similarity of two sets which takes values on the interval [0, 1] and has all the properties associated with a true metric. It is defined as the cardinality of the intersection of the two sets divided by the cardinality of their union.

(10) $$J_{AB}=\frac{\left|A\cap B\right|}{\left|A\cap B\right|}$$

In addition, to the above, Table 7 and Table 8 also include the number of SCOP groups or clusters found at a given level, the number of the unique, most similar groups to which these are mapped as well as the ratio between these numbers minus one, which provides a readable expression of the closeness of the match.

We selected several PSS representations to subject to this cluster analysis. These include pairwise MATT probability scores as well as the top-performing MPSS constructed using SMACOF on the Dali and MATT raw scores. In order investigate the impact of varying MPSS dimensionality on structure space clustering we include the minimum and maximum dimensionalities found on the AUC performance plateau observed in Figure 7 and 8. This corresponds to dimensionalities of 12 and 30 for Dali and 12 and 24 for MATT.

**Figure 10:**
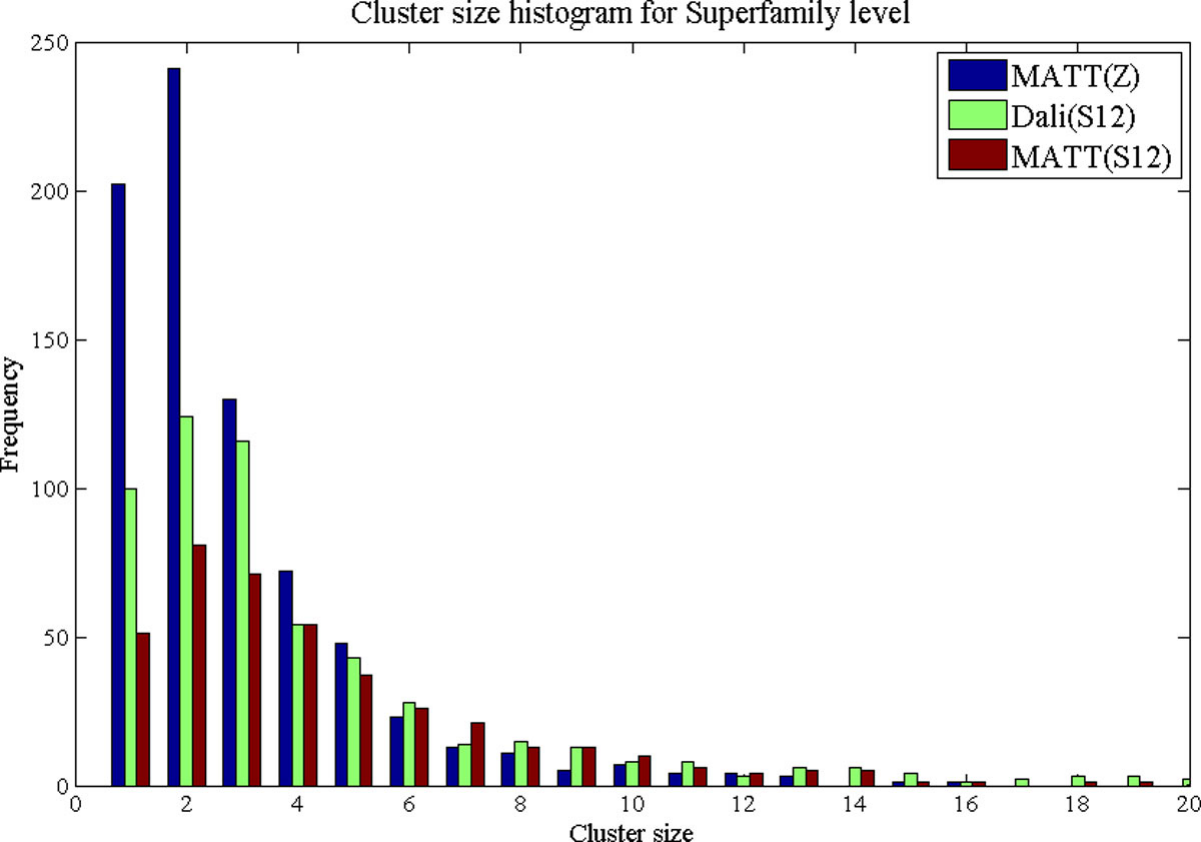
**Histogram of cluster sizes for SCOP Superfamily level**. Frequency of occurrence is plotted versus the size of clusters obtained by hierarchical clustering using the Superfamily-level distance threshold. Pairwise MATT distances result in a large number of small clusters, including many singletons. For MPSS, in comparison, a smaller number of larger clusters which map more directly to the SCOP Superfamily classification are obtained.

**Figure 11:**
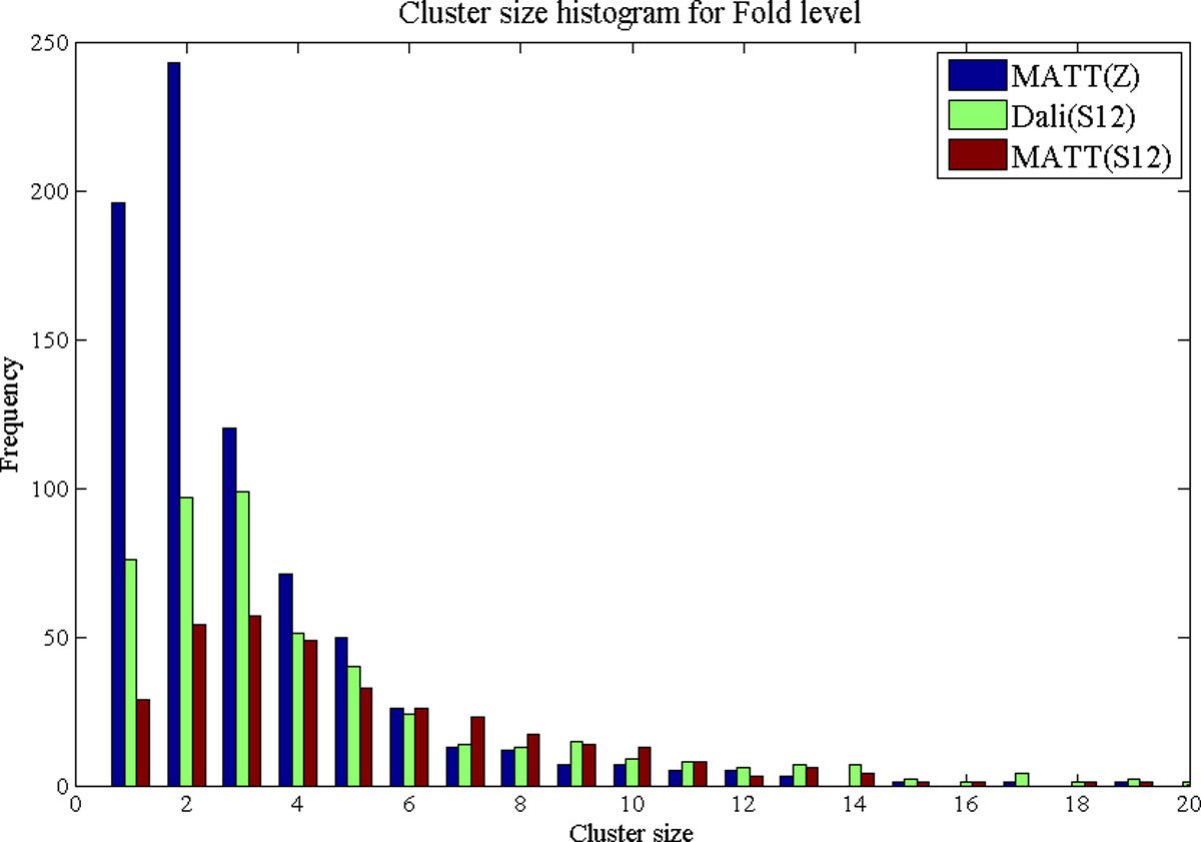
**Histogram of cluster sizes for SCOP Fold level**. The frequency of occurrence is plotted against the size of clusters obtained by hierarchical clustering using the Fold-level distance threshold. As for the SCOP Superfamily level, pairwise MATT distances result in a large number of small clusters, including many singletons. In comparison, for MPSS, a smaller number of larger clusters which map more directly to the SCOP Fold classification are obtained.

The results of Table 7 and Table 8 reveal that MATT cleaves SCOP Superfamilies into many more clusters than do the MPSS distances. This leads both to a very high maximum and standard deviation of splitting degree. The closest correspondence between individual SCOP clades and hierarchical clusters is achieved by 12D MPSS using either Dali or MATT. Although SCOP clades map more readily to the automatic clusters than vice-versa, these same trends are nevertheless evident in the bottom half of Table 7 and Table 8. The situation appears somewhat different when the actual similarities (Jaccard indices) are examined in addition to the splitting degree. Although MATT is less consistent than the MPSS (greater standard deviation), it seems to achieve a slightly greater level of similarity between clusters and their matching clades in SCOP. Interestingly, the same observation holds between the relative high- and low-dimensional MPSS. Why is this given the significantly worse performance of these PSS representations in terms of cluster integrity? The answer is given by the relatively large number of small SCOP groupings, including many singletons, present in the data set used in this work. The better mean Jaccard coefficients arise merely because clustering of these PSS representations leads to a large number of similarly small clusters including a large number of singletons with high chance similarity to the singleton SCOP clades. This interpretation is supported by the fact that MATT and high-dimensional MPSS have worse (less consistent) standard deviations than the low-dimensional MPSS, and by the very large numbers of singleton and small clusters observed for MATT in Figure 10 and 11, which display histograms of cluster size for MATT and the 12D MPSS. The reader may also note that this relationship between high- and low-dimensional MPSS is expected even when the accuracy of pairwise classification (AUC) is very similar, as is the case for 12D and 30D Dali and MATT MPSS. The reason for this is the "curse of dimensionality" commonly lamented in clustering applications. Because distance between points increases monotonically with dimensionality, so will the number of splits in the dendrograms obtained by neighbor-joining. This leads in turn to a greater number of final clusters and a greater chance of finding singletons. Note that the higher distance thresholds appropriate for higher dimensionalities are supplied automatically by the ROC curve threshold selection procedure, thus the greater number of clusters is due only to the changes to the topology of the tree which guides the maximum-linkage clustering.

## Conclusions

We have presented an extensive investigation of structure space representations. In particular we have analyzed the biological relevance of 24 such representations in terms of their capacity for automatic classification of SCOP Superfamilies and SCOP Folds, as well as the similarity found between automatic tessellation of PSS representations and the clade systems in SCOP. These representations use four state-of-the-art structure alignment algorithms, CE, Dali, FATCAT and MATT, which exemplify divergent approaches to the alignment problem, and separate comparisons were made using both raw alignment distances and the transformed alignment distances which attempt to account for alignment length and statistical significance.

It was found that all but one of these transformations were less effective than projection into an explicit spatial representation using MDS, despite the fact that MDS makes no use of alignment length or structure size and requires only a single, insensitive parameter (the target dimensionality). We also examined the impact of dimensionality on the accuracy of MPSS. This is an important addition to our previous work investigating parameters involved in creation of MPSS [13]. The results indicate that MPSS are highly effective for dimensionalities ranging between approximately 12 and 30, although they remain competitive with pairwise distances even for the very low-dimensionalities suitable for visualization. In particular, a 12D MPSS generated by projection of pairwise Dali distances using the SMACOF algorithm for MDS is able to predict the membership of protein pairs in SCOP Superfamiles and SCOP Folds with respective AUC values of 0.942 and 0.904. These values are higher than those for any of the other 23 types of intermolecular distances examined here. Finally, we tested how similar automatic clustering of the best-performing PSS representations can be to the tessellation of protein space defined by the Superfamily and Fold levels of SCOP. Using a hierarchical clustering algorithm parameterized by a single distance threshold, MPSS permit a tessellation of the PSS which is remarkably similar to SCOP at both of the remotely homologous levels considered here.

We thus conclude that MPSS constructed by multidimensional scaling of pairwise distance data provide a powerful framework for automated analysis of structure-function relationships between proteins. It is particularly noteworthy that MPSS are highly effective even at the very remotely homologous Superfamily and Fold levels of SCOP. Future directions of our research will focus on the usefulness of the explicit coordinate representation of the protein fold space, in order to address the fundamental character of the protein fold space, as well as the specific regions in that space which correspond to particular types of structures or functional capacities.

## List of abbreviations

SCOP: Structural Classification of Proteins; CATH: Class, Architecture, Topology, Homology; MPSS: Map(s) of Protein Structure Space; PDB: Protein Data Bank; CE: Combinatorial Extension (of the optimal path); FATCAT: Flexible structure AlignmenT by Chaining Aligned fragment pairs allowing Twists; MATT: Multiple Alignment allowing Twists and Turns; MDS: Multidimensional Scaling; PSS: Protein Structure Space; AFP: Aligned Fragment Pair; RMSD: Root Mean Square Deviation; CMDS: Classical Multidimensional Scaling; SMACOF: Scaling by Majorizing a Complex Function; ROC: Receiver Operating Characteristic; FPR: False Positive Rate; TPR: True Positive Rate; AUC: Area Under the Curve; TCR: True Classification Rate; TNR: True Negative Rate; NJ: Neighbor Joining; PH: Pleckstrin Homology; NTF2: Nuclear Transport Factor 2.

## Competing interests

The authors declare that they have no competing interests.

## Authors' contributions

R. Singh conceived the research formulation. The algorithmic and experimental design was done by D. Asarnow and R. Singh. D. Asarnow implemented methods and analyzed the data. Both authors contributed to the composition of the manuscript.

## Declarations

This work and publication costs for the article were supported by the National Science Foundation grant IIS0644418 (CAREER).

This article has been published as part of *BMC Bioinformatics *Volume 15 Supplement 2, 2014: Selected articles from the Twelfth Asia Pacific Bioinformatics Conference (APBC 2014): Bioinformatics. The full contents of the supplement are available online at http://www.biomedcentral.com/bmcbioinformatics/supplements/15/S2.
